# Sex-related differences in the efficacy of Baclofen enantiomers on self-administered alcohol in a binge drinking pattern and dopamine release in the core of the nucleus accumbens

**DOI:** 10.3389/fphar.2023.1146848

**Published:** 2023-03-16

**Authors:** Jérôme Jeanblanc, Pierre Sauton, Charles Houdant, Sandra Fernandez Rodriguez, Sofia Vilelas de Sousa, Virginie Jeanblanc, Sandra Bodeau, Laurence Labat, Marion Soichot, Florence Vorspan, Mickael Naassila

**Affiliations:** ^1^ INSERM UMR-S 1247, Research Group on Alcohol and Pharmacodependences (GRAP), Université de Picardie Jules Verne, Amiens, France; ^2^ Animal Facility of the Université de Picardie Jules Verne, Amiens, France; ^3^ MP3CV Laboratory, Department of Clinical Pharmacology, Amiens University Hospital, University of Picardie Jules Verne, Amiens, France; ^4^ INSERM UMR-S 1144, Optimisation Thérapeutique en Neuropsychopharmacologie, Université de Paris, Paris, France; ^5^ Faculté de Médecine, Université de Paris Cité, Paris, France; ^6^ Département de Psychiatrie et de Médecine Addictologique, Hôpital Fernand Widal, Assistance Publique—Hôpitaux de Paris, Paris, France; ^7^ Laboratoire de Toxicologie Biologique, Hôpital Lariboisière, Paris, France; ^8^ INSERM UMRS1144, Département de Psychiatrie et de Médecine Addictologique, Assistance Publique—Hôpitaux de Paris, GH Lariboisière—Fernand Widal, GHU NORD, Université de Paris, Paris, France; ^9^ GDR3557 Psychiatrie-Addictions, Institut de Psychiatrie, University Hospital Federation (FHU A2M2P), Caen, France

**Keywords:** binge drinking, Baclofen, enantiomers, sex, dopamine, alcohol, rat

## Abstract

**Introduction:** Clinical studies on the effectiveness of Baclofen in alcohol use disorder (AUD) yielded mixed results possibly because of differential effects of the enantiomers and sex-related differences. Here we examined the effect of the different Baclofen enantiomers on alcohol intake and on evoked dopamine release in the core of the nucleus accumbens (NAcc) in male and female Long Evans rats.

**Methods:** Rats were trained to chronically self-administer 20% alcohol solution in daily binge drinking sessions and were treated with the different forms of Baclofen [RS(±), R(+) and S(−)]. The effects on the evoked dopamine release within the core of the nucleus accumbens were measured in brain slices from the same animals and the alcohol naïve animals using the fast scan cyclic voltammetry technique.

**Results:** RS(±)-Baclofen reduced alcohol intake regardless of sex but more females were non-responders to the treatment. R(+)-Baclofen also reduced alcohol intake regardless of sex but females were less sensitive than males. S(−)-Baclofen did not have any effect on average but in some individuals, especially in the females, it did increase alcohol intake by at least 100%. There were no sex differences in Baclofen pharmacokinetic but a strong negative correlation was found in females with a paradoxical effect of increased alcohol intake with higher blood Baclofen concentration. Chronic alcohol intake reduced the sensitivity to the effect of Baclofen on evoked dopamine release and S(−)-Baclofen increased dopamine release specifically in females.

**Discussion:** Our results demonstrate a sex-dependent effect of the different forms of Baclofen with no or negative effects (meaning an increase in alcohol self-administration) in subgroup of females that could be linked to a differential effect on dopamine release and should warrant future clinical studies on alcohol use disorder pharmacotherapy that will deeply analyze sex difference.

## 1 Introduction

Alcohol use disorder (AUD) is a chronic disease associated with high rates of mortality and morbidity. Several AUD treatments are currently available with modest effect sizes for efficacy. For example, the numbers needed to treat (NNT) for benefit regarding the return to any drinking endpoint have been calculated to 11 and 20 for acamprosate and naltrexone, respectively (Jonas et al., 2014). The NNT estimates the number of patients that need to be treated in order to have an impact on one patient. Medications are used either to maintain abstinence [disulfiram, acamprosate, naltrexone, gamma-hydroxybutyrate, and RS(±)-Baclofen] or to reduce drinking [nalmefene and RS(±)-Baclofen].

Among the AUD pharmacotherapies, RS(±)-Baclofen [(±)-4-amino-3-(p-chlorophenyl)-butanoic acid], the racemic form, a high affinity γ-aminobutyric acid type B (GABA_B_) receptor agonist can increase rates of abstinence and reduce alcohol craving and anxiety. The RS(±)-Baclofen (30–75 mg) has been shown to reduce both alcohol cue reactivity in prefrontal brain regions and in the percentage of heavy drinking days, but with no changes in craving (Logge et al., 2019). The RS(±)-Baclofen obtained a “Temporary Recommendations for Use” in AUD in 2014 and a Marketing Authorization Approval for doses up to 80 mg daily in 2018 (Rolland et al., 2020). Different studies on alcohol abstinence suggested that RS(±)-Baclofen is not better than placebo (Agabio et al., 2018; Minozzi et al., 2018; Rose and Jones, 2018). Other studies have reported concerns about adverse effects of RS(±)-Baclofen especially at high doses and when taken with alcohol (Rolland et al., 2014, 2015). Side effects of Baclofen also contributed to its limited clinical use and may have participated to the controversy. Preclinical studies demonstrated clear sedative effects and clinical studies reported important side effects including sedation, drowsiness and sleepiness. Controversial data about RS(±)-Baclofen efficacy in AUD may come from numerous criteria such as the optimal dose, highly variable plasma levels of RS(±)-Baclofen (Chevillard et al., 2018; Simon et al., 2018), levels of comorbid anxiety, outcomes (abstinence vs. intake reduction), lack of regarding the responder characteristics, genetics and the severity of the disease (Marsot et al., 2014; Morley et al., 2014; Durant et al., 2018; Farokhnia et al., 2018).

The RS(±)-Baclofen efficacy to decrease alcohol intake in rats has been demonstrated for a long time (Daoust et al., 1987; Colombo et al., 2000, 2005; Anstrom et al., 2003; Janak and Michael Gill, 2003; Stromberg, 2004), but at very variable doses, generally ranging from 1 to 10 mg/kg on ethanol intake or even 40 mg/kg on withdrawal signs (Colombo et al., 2000). Numerous alcohol-related behaviours (acquisition of alcohol drinking and self-administration, seeking reinstatement, relapse-like drinking) were reduced by Baclofen (Haile et al., 2021). An increase in alcohol intake has also been demonstrated after RS(±)-Baclofen treatment in two-bottle choice and operant procedures (Smith et al., 1992; Petry, 1997; Smith et al., 1999). For example, RS(±)-Baclofen reduced alcohol and sucrose responding at 5 mg/kg but increased alcohol and decreased sucrose responding at 1.25 mg/kg (Petry, 1997). The effect of Baclofen may be mediated by changes in dopamine transmission of the brain reward circuit. For example, the dopamine release in the core of the nucleus accumbens (NAcc) was inhibited by R(+)-Baclofen (Pitman et al., 2014).

It is the racemic compound that is used in patients. The racemic compound breaks down into absolute configurations of R- and S- and positive (+) and negative (−) molecular rotations. There were fewer studies on the effects of the two enantiomers S(−)-Baclofen and the R(+)-Baclofen. Enantiomers are molecules that are mirror images of each other. The biological action of the racemic compound is known to reside in the active R(+)-Baclofen (Olpe et al., 1978). The IC_50_ values for R(+)-Baclofen, S(−)-Baclofen, and racemic Baclofen for the inhibition of binding of [^3^H]-Baclofen to GABA receptors of cat cerebellum are 15 nM, 1.77 μM, and 35 nM, respectively (Froestl et al., 1995).

R(+)-Baclofen reduced alcohol intake, motivation to consume alcohol and alcohol relapse in a relevant animal model of binge self-administration (González-Marín et al., 2018; Jeanblanc and Rolland, 2018; Jeanblanc and Sauton, 2018). In the post-dependent model of AUD R(+)-Baclofen was also more effective than RS(±)-Baclofen in reducing alcohol intake and seeking during acute withdrawal and during relapse after abstinence in male rats (Echeverry-Alzate et al., 2020). Both S(−)-Baclofen and RS(±)-Baclofen, but not R(+)-Baclofen, increased alcohol intake in a subpopulation of rats, thus highlighting a wide variability in the therapeutic responses depending on the enantiomers (Echeverry-Alzate et al., 2020). Altogether these data suggested that R(+)-Baclofen may be the most promising enantiomer; however, no data are available regarding sex-related difference in the efficacy of the different enantiomers and there are also no data regarding the effect of these enantiomers on the modulation of dopamine signaling within the NAcc, the key brain structure involved in the rewarding effects of alcohol.

Because there are no preclinical data and only one clinical study that suggested sex-related difference in RS-Baclofen response, we conducted experiments in both male and female outbred Long Evans rats. In addition, since the efficacy may be linked to pharmacokinetic factors (wide interindividual variability) (Marsot et al., 2014), we quantified plasmatic Baclofen concentrations to correlate them to the efficacy of the drugs. Finally, we used the *ex vivo* fast-scan cyclic voltammetry technique in order to measure the effects of the different enantiomers on dopamine signaling in the core of the NAcc.

## 2 Materials and methods

### 2.1 Reagents and drug injections

Alcohol was purchased from WWR (Prolabo, Fontenay-sous-Bois, France) and diluted into tap water at a 20% concentration (v/v) for the behavioral study or in artificial cerebro-spinal fluid (aCSF) at the concentration of 100 µM for the fast-scan cyclic voltammetry experiment. NaCl, KCl, NaH_2_PO_4_, MgCl_2_, CaCl_2_, NaHCO_3_, glucose and ascorbic acid were purchased from Sigma-Aldrich (Saint Quentin Fallavier, France). R(+)-Baclofen, S(−)-Baclofen and RS(±)-Baclofen were obtained from (Sigma Aldrich, Saint Quentin Fallavier, France). For the behavioral experiments, all drugs were dissolved in 0.9% sterile saline. Drugs were i.p. administered 30 min before the start of the operant alcohol self-administration sessions. Baclofen’s enantiomers were administered at different doses, 1.5 mg/kg for the first experiment and 0.5, 1.0, 1.5, 2 or 3 mg/kg, in a volume of 1 mL/kg of body weight, subsequently. All solutions were used at room temperature, and dose and routes of administration were chosen accordingly to our previous works (González-Marín et al., 2018; Echeverry-Alzate et al., 2020). Regarding the fast-scan cyclic voltammetry experiment, Baclofen’s enantiomers were used at a concentration of 100 µM in aCSF (Pitman et al., 2014).

### 2.2 Animals

Long Evans male (*n* = 32) and female (*n* = 37) rats were obtained from Janvier Laboratories (Le Genest-Saint-Isle, France) at the age of 7 weeks. After a week of habituation, rats weighting an average body weight of 260 ± 20 g in males and 180 ± 10 g in females started the experimental protocol. Rats were housed in 365 mm × 265 mm × 230 mm plastic isolated ventilated cages (1 rat per cage) with Lab cob 12 bedding (Serlab, Montataire, France) in a temperature- (21°C ± 1°C) and humidity-controlled (30%–70%) environment with a 12-h light (7:00–19:00)/dark cycle, with free access to food diet n°3436 (Serlab, Montataire, France) and water. All experiments were performed between 2:00 p.m. and 5:00 p.m.

A first cohort of 19 rats (10 females and 9 males) was used for the dose responses experiments with the 3 forms of Baclofen. We chose a within-subject design as already published (González-Marín et al., 2018; Jeanblanc and Sauton, 2018). In this cohort, animals received the R(+)-Baclofen (3 doses in a random order, each dose was tested after at least a 2-day washout period). We previously showed that a total recovery of basal levels of alcohol self-administration is observed with Baclofen after a 2-day washout period (González-Marín et al., 2018; Jeanblanc and Sauton, 2018). One week after the last dose, they received S(−)-Baclofen (4 doses in a random order). Finally, 1 week after the last dose they received RS-Baclofen (5 doses in a random order).

A second cohort of 30 animals (15 females and 15 males) was used to test the individual variability of the dopamine efflux at the 1.5 mg/kg dose of the 3 forms of Baclofen. In this cohort, animals received all doses of the 3 forms of Baclofen in a random order. We also measured blood levels of Baclofen after injection of 1.5 mg/kg RS-Baclofen in all animals, in order to look for correlations between Baclofen effectiveness and its blood levels.

A third cohort of 20 animals (12 females and 8 males) was used, and not exposed to alcohol, for the measurements of Baclofen and its metabolites to investigate the sex effects after injection of the 3 different forms of Baclofen at the dose of 1.5 mg/kg.

### 2.3 Ethical statement

All of the experiments were submitted for prior approval to the local ethical committee (CREMEAP: Comité Régional d'Ethique en Matière d'Expérimentation Animale de Picardie) and validated by the French Ministry in charge of the Research under the number #2145-201510051547534v2. All experiments were performed in conformity with the European Community guiding principles for the care and use of animals (2010/63/UE, CE Off. J. 20 October 2010), the French decree n° 2013–118 (French Republic Off. J., 2013). All the procedures used were declared to and approved by the local animal welfare structure (Structure du Bien Etre Animal, SBEA).

### 2.4 Blood concentrations of Baclofen and metabolites

Blood collection was done on a separate day 30 and 90 min after the i.p. injection of the RS(±)-Baclofen 1.5 mg/kg). Rats were anesthetized under isoflurane (5% for 2 min) and blood was collected in heparinized tubes (±200 μl) from the sublingual vein. Samples were centrifuged and stored on ice. Blood levels of Baclofen (ng/mL) were determined by HPLC (High Performance Liquid Chromatography).

Baclofen and its deaminated metabolite [M1, 3-(4-chlorophenyl)-4-hydroxybutyric acid] were determined by liquid chromatography (Shimadzu, Marne-la-Vallée, France) coupled to a tandem mass spectrometer (3200Qtrap, Sciex, Les Ulis, France). Briefly, rats were i.p. injected with either one of the 3 forms of Baclofen at the dose of 1.5 mg/kg. Blood was collected 30 and 240 min after the injection using the same sublingual technique than described above. Then, Baclofen and its metabolite were extracted from 50 µL of plasma by adding 250 µL of an iced acetonitrile solution containing the internal standard (baclofen-d4) at 100 ng/mL. After centrifugation, 250 µL of the supernatant was evaporated to dryness and then taken up by 100 µL of a mixture acetonitrile/water (10/90, v/v) before transfer to a vial for injection into the chromatographic system. Chromatographic separation was performed at 40°C on an ultra PFP Propyl column (5 μm, 50 mm × 2.1 mm, Restek, Lisses, France). The column was eluted with a gradient of acetonitrile with 0.1% formic acid and ultra-pure water with 0.1% formic acid delivered at a flow rate of 0.5 mL/min. Data were acquired in multiple reaction monitoring mode after ionization in positive (for the Baclofen) or negative (for M1) electrospray ionization modes. We also measured Baclofen and its metabolites after an injection of 3 mg/kg RS-Baclofen in order to get a result with a 1:1 ratio of both enantiomer (1.5 mg/kg of each enantiomer). We did not see any sex differences in this latter experiment (data not shown).

### 2.5 Self-administration of alcohol

Rats were trained to self-administer alcohol (0.1 mL of a 20% alcohol solution v/v per delivery) in a binge drinking pattern, as described previously (González-Marín et al., 2018; Jeanblanc and Sauton, 2018). Briefly, rats underwent a 2-bottle-choice 20% intermittent access paradigm for 4 weeks before the self-administration sessions. The schedule of self-administration sessions was: 2 overnight sessions under a fixed ratio 1 schedule (FR1), FR1—1 h for 5-7 sessions, FR3—1 h for 5-7 sessions, FR3—30 min for 5-7 sessions and finally FR3-15 min until stable baseline is reached. All sessions (except the overnight ones) were conducted between 2:00 p.m. and 5:00 p.m. This reduction in the duration of the sessions led to intoxicating levels of alcohol self-administration (Jeanblanc and Sauton, 2018). The number of active and inactive lever presses as well as the number of reinforcers obtained were recorded during each operant self-administration session. A within-subjects design was used in which each rat was its own control and thus received the different doses of the enantiomers in random order. Washout sessions were performed between each new session of drug test, as previously described (Echeverry-Alzate et al., 2020).

### 2.6 Mesolimbic phasic dopamine transmission: Fast-scan cyclic voltammetry (FSCV)

Rats were euthanized (deep anesthesia with Isoflurane 5% and decapitation) and coronal slices containing the NAcc were collected to measure electrically evoked dopamine transmission in the core of the NAcc (Figure 4 upper panels). Coronal slices corresponding to the Paxinos and Watson atlas (1998) slices from the +1.60 to +1.00 mm from Bregma were used for this study. Recording electrodes were implanted below the anterior commissure and the stimulating electrode 100 µm below the recording electrode. Rats were anesthetized with isoflurane (IsoVet, 5%) before being decapitated, and their brain were extracted and immersed in ice-cold artificial cerebrospinal fluid (aCSF) (NaCl 126 mM, KCl 2.5 mM, NaH_2_PO_4_ 1.1 mM, MgCl_2_ 1.4 mM, CaCl_2_ 0.5 mM, NaHCO_3_ 18 mM, Glucose 11 mM, ascorbic acid 0.4 mM, pH 7.2–7.4) and glued into a vibratome (Leica, VT 1200S). Coronal slices (250 µm thick) of the NAcc were selected and stored in a 31°C aCSF (NaCl 126 mM, KCl 2.5 mM, NaH_2_PO_4_ 1.1 mM, MgCl_2_ 1.4 mM, CaCl_2_ 2.4 mM, NaHCO_3_ 18 mM, Glucose 11 mM, pH 7.2–7.4) reservoir gassed with carbogen (95% O_2_, 5% CO_2_) for at least 1 h. After rest, the slices were transferred to a recording chamber and perfused with aCSF (3 ml/min, 31°C). Then, slices were placed in a recording chamber and were continuously superfused with an aCSF solution containing 2.4 mM CaCl_2_ saturated with 95% O_2_ and 5% CO_2_. FSCV using carbon-fiber microelectrodes (7 μm diameter; 125 μm exposed surface; Goodfellow, Cambridge, United Kingdom) was used to detect extracellular DA concentration. The scan rate was 400 V/s with a sampling interval of 100 ms and the scan range was from −0.4 to +1.3 V (vs. Ag/AgCl). DA release was evoked by a single stimulation of 300 µA for 0.5 ms every 5min. The concentration of the extracellular DA was calculated based on a standard curve (0.1, 1, and 10 μM DA) obtained for each microelectrode at the end of each recording session.

All analyses of release and uptake were conducted on the concentration-*vs.*-time traces. These traces were fit to a model describing dopamine signaling as a balance between release and uptake, using the Michaelis-Menten equation [see(Wu et al., 2001)], with the Lvit software (Scott Ng-Evans, Electronics and Materials Engineering Shop, Seattle, WA, United States). The equation was as follow:

$$d\left[DA\right]/dt=f{\left[DA\right]}_{p}-V_{\max}/\left(\left(K_{m}/\left[DA\right]\right)+1\right)$$

Where [DA] is the instant extracellular concentration of DA released, f is the frequency of stimulation, [DA]_p_ is dopamine concentration released per pulse, and V_max_ and K_m_ were respectively the velocity and affinity constants of the dopamine transporter (DAT). K_m_ was fixed at a constant value of 200 nM (Wu et al., 2001). [DA]_p_ reflects presynaptic mechanisms regulating release as those of the auto-receptors (D2/D3 activity) (Kennedy et al., 1992). Peak dopamine concentration was extracted for each trace before fitting to the model in order to obtain a [DA]_max_ value, reflecting maximum extracellular dopamine concentration, and was used as a parameter of dopamine release. [DA]_p_ and V_max_ values were modulated until fitting the traces to the model, with a correlation coefficient of 0.8 or more with our experimental data, using the Lvit software.

### 2.7 Statistical analysis

Statistical analysis was done only on datasets of *n* ≥ 5. Appropriate sample sizes using the expected variance and effect size were estimated from the previous experiments using similar methods (González-Marín et al., 2018; Jeanblanc and Sauton, 2018). Animals were randomly allocated to each experimental group. In general, the group sizes for each experiment are provided within the figure legends. All experiments were performed in a blinded manner in order to limit personal bias. The SigmaPlot 11.0 (Systat Software, Inc.) and Prism 8 (GraphPad) softwares were used for all statistical analyses. Data are expressed as Mean ± SEM and analyzed with an ANOVA (1- or 2-way) with repeated measures. Multiple comparisons were performed using the Tukey test. A Pearson test was used for the correlation studies. For non-parametric analysis (inactive lever presses) a Kruskal-Wallis test was used followed by a Dunn’s Multiple Comparison Test. The significance of the analysis was set to 0.05. When not significant, probability of the test is summarized “ns”.

## 3 Results

### 3.1 Effects of the different forms of Baclofen on alcohol self-administration

**FIGURE 1:**
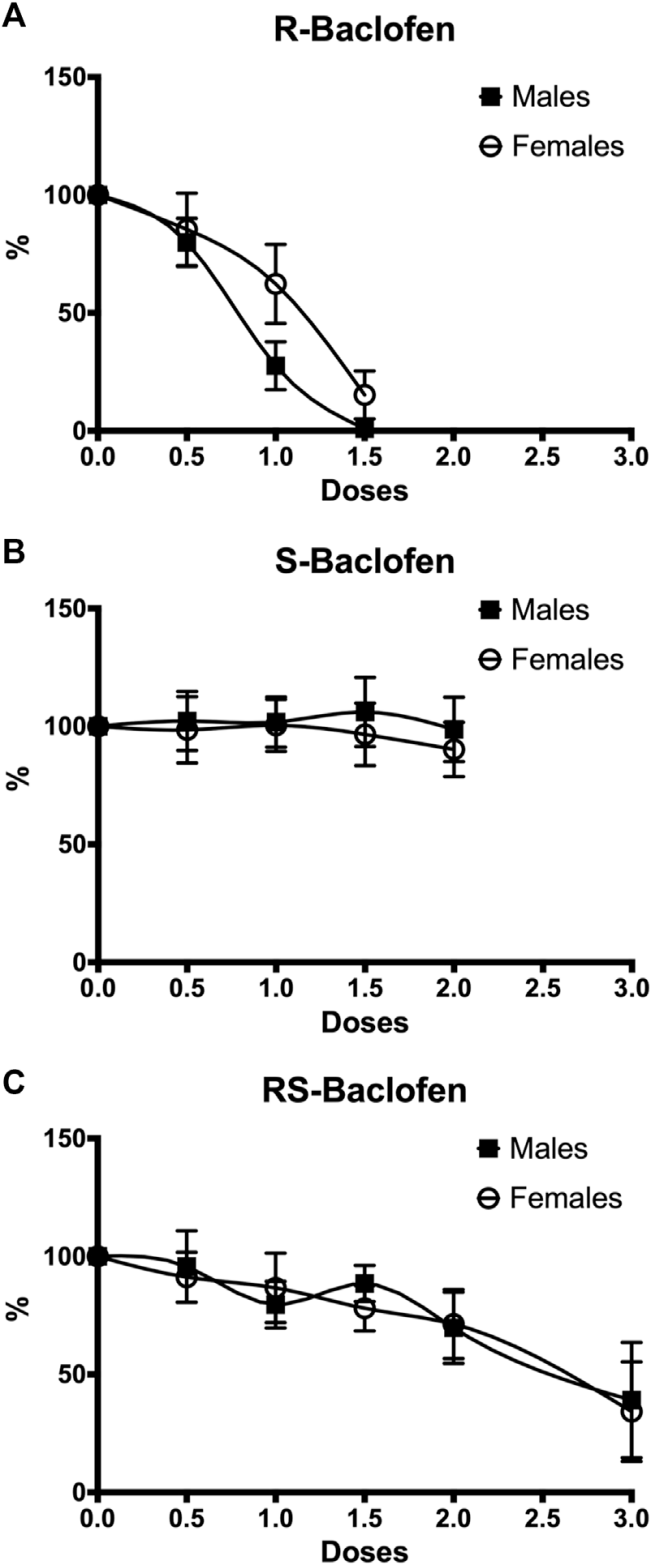
Dose-response effects on alcohol intake in both males (*n* = 9) and females (*n* = 10) for R(+)-Baclofen **(A)**, S(−)-Baclofen **(B)** and RS-Baclofen **(C)**. All doses of the different forms of Baclofen were administered in a random order. Rats received first the R(+)-Baclofen, then the S(−)-Baclofen and finally the RS-Baclofen. A significant dose and sex effect was found only for the R(+)-Baclofen. Data have been normalized so that the 100% represents the level of ethanol self-administration during the last session of the 5 consecutive sessions (days) of habituation (saline but no baclofen injection).

In a first cohort of 9 males and 10 females, dose-responses for the 2 enantiomers and the racemic form have been generated and results are presented in Figure 1. For the R(+)-Baclofen analysis, the 2-way RM ANOVA revealed a significant effect of the factor dose (F_(3,51)_ = 6.61, *p* < 0.0001) and of the factor sex (F_(1,17)_ = 0.77, *p* = 0.046) and showed no interaction between these factors (F_(3,51)_ = 0.06, *p* = 0.83). For the S(−)-Baclofen analysis, the 2-way RM ANOVA revealed no significant effect of the factor dose (F_(4,64)_ = 0.19, *p* = 0.94) and of the factor sex (F_(1,16)_ = 0.16, *p* = 0.69) and showed no interaction between these factors (F_(4,64)_ = 0.10, *p* = 0.97). For the RS(±)-Baclofen analysis, the 2-way RM ANOVA revealed a significant effect of the factor dose (F_(5,80)_ = 7.01, *p* < 0.0001) but not of the factor sex (F_(1,16)_ = 0.02, *p* = 0.87) and showed no interaction between these factors (F_(5,80)_ = 0.12, *p* = 0.98).

**FIGURE 2:**
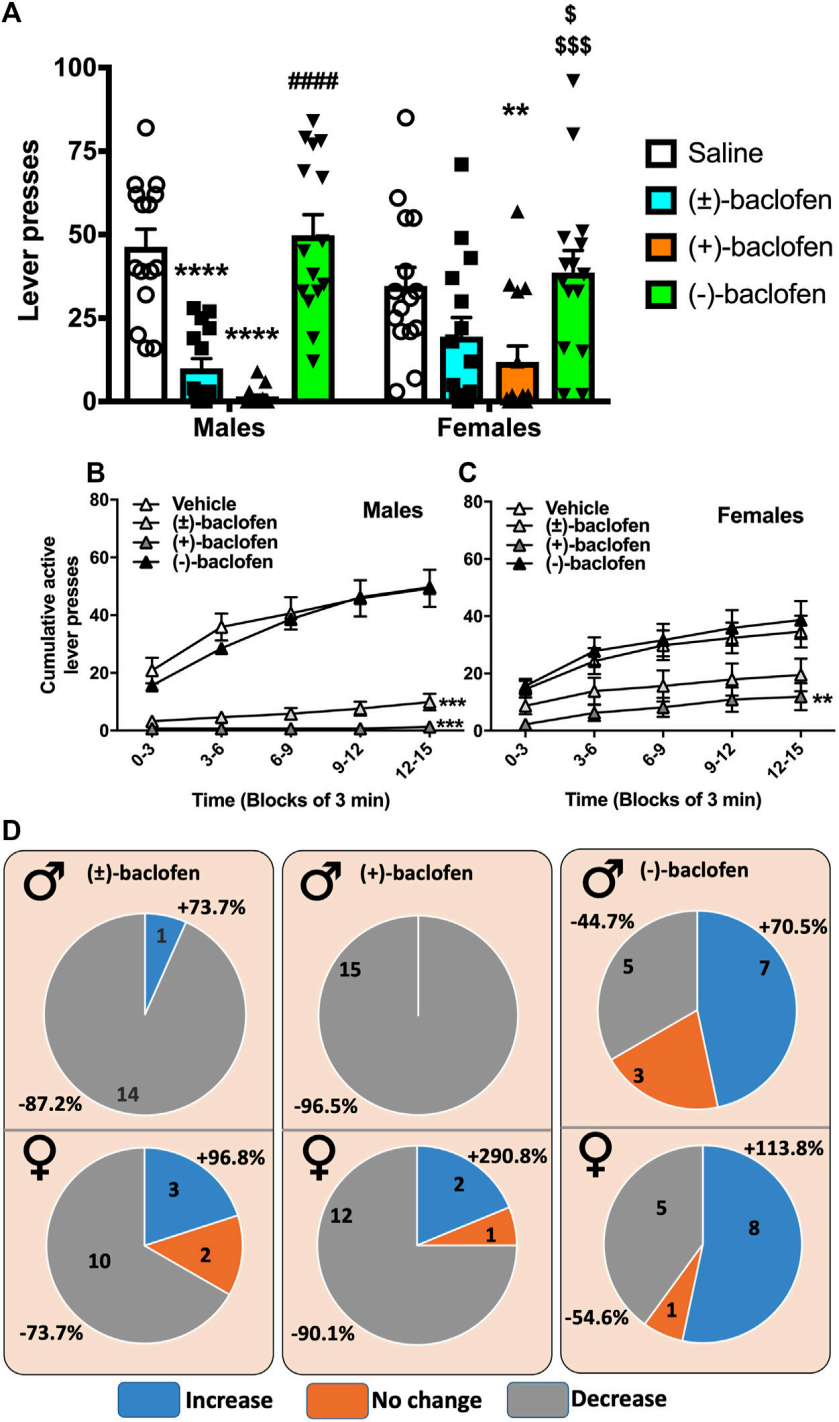
Effect of the different forms of Baclofen on alcohol operant self-administration in male and female rats. **(A)** The RS(±)-, the R(+)- and the S(−)-Baclofen were administered i.p. at the dose of 1.5 mg/kg 30 min prior to a 15-min session of alcohol self-administration. Each rat was its own control and received the 4 injections in random order. Results are expressed as Mean ± SEM of pure alcohol consumed in g/kg. *p < 0.05, **p < 0.01, ***p < 0.001, **** *p* < 0.0001, #### *p* < 0.0001 vs. R (+) and RS (±)-Baclofen, and $$$$ *p* < 0.0001 vs. RS(±)-Baclofen. **(B)** Cumulative lever presses observed after the injection of the different forms of Baclofen in male rats. Results are expressed as Mean ± SEM of the cumulative presses in 3-min bins over the 15 min session. ***p < 0.001 vs. Vehicle. **(C)** Cumulative lever presses observed after the injection of the different forms of Baclofen in female rats. Results are expressed as Mean ± SEM of the cumulative presses in 3-min bins over the 15 min session. **p < 0.01 vs. Vehicle. **(D)** Distribution of animal responses regarding sex (M for males and F for females on the left corner) and the treatments. Numbers inside the circle indicate the number of animals over the total of 15 rats. Percentages outside the circle express the amplitude of the variation in alcohol self-administration as compared to the vehicle treatment. Light grey represents a decrease in alcohol self-administration. The mild grey represents no change (between (−10 and +10%) as compared to vehicle. The dark grey represents an increase in alcohol self-administration as compared to vehicle. The results depicted in each of these 4 panels were provided by the same cohort of rats, 15 males and 15 females.

In a second cohort, the 3 forms of Baclofen were tested at the dose of 1.5 mg/kg vs. a saline injection and the results are depicted in Figure 2. We found that the S(−)-Baclofen had no effect on the average levels of alcohol consumed. The RS(±)-Baclofen at this dose and in this cohort was more efficacious in decreasing alcohol self-administration in males than in females. Regarding R(+)-Baclofen, it was more efficacious than the racemic form in both sexes.

The analysis of the self-administered ethanol expressed as the number of active lever presses (Figure 2A) with a 2-way RM ANOVA, revealed no effect of the factor sex (F_(1,28)_ = 0.29, *p* = 0.86), a significant effect of treatment (F_(3,84)_ = 30.24, *p* < 0.0001) and also a significant interaction between those factors (F_(3,84)_ = 3.35, *p* = 0.022). The *post hoc* analysis revealed significant differences between groups. Within the males, the Tukey test indicated a significant difference between the Saline group and both the RS(±)-Baclofen (*p* < 0.0001) and R(+)-Baclofen (*p* < 0.0001). The S(−)-Baclofen was significantly different from the R(+)- and the RS(±)-Baclofen (*p* < 0.0001) but not from the Saline group. Regarding the females, from the 3 forms of Baclofen, only the R(+) showed a significant difference from the Saline group (*p* = 0.006) and from the S(−)-Baclofen group (*p* = 0.0009). This S(−)-Baclofen group was also significantly different from the RS(±)-Baclofen group (*p* = 0.029). Between the sexes, the males displayed a significantly larger effect than the females with the RS(±)-Baclofen. Results were similar when analyzed by the self-administered ethanol expressed as g of pure ethanol per kg of body weight (Supplementary Figure 1A). Regarding inactive lever presses, only a moderate decrease in responding was observed after the R(+)-Baclofen injection in the males (Supplementary Figure 1B). But the response levels were already so low in average that this decrease was difficult to interpret. In the female group, no difference was observed for the numbers of inactive lever presses (Supplementary Figure 1C).

Patterns of self-administration in males and females are shown on Figures 2B, C and no statistical analysis is provided because of the nature (cumulative) of the data. In both sexes, the R(+)- and the RS(±)-Baclofen groups seemed different (lower cumulative responses) from both the Saline and the S(−)-Baclofen groups. Sex differences were observed by analyzing the inter-response intervals (IRI, Supplementary Figures 2A, B). In the Males group, we observed a decrease in the proportion of IRI within the (0, 1 s) interval for both the R(+) and the RS(±)-Baclofen (*p* < 0.001, Supplementary Figure 2A) and an increase of the proportion for the interval (1, 2 s) for the R(+)-Baclofen as compared to the Saline, the RS(±)- and the S(−)-Baclofen groups (*p* < 0.001). The longest IRI (20 s and more) were also increased in the males group after the administration of R(+) and RS(±)-Baclofen (*p* < 0.001). On the contrary, in the Females group (Supplementary Figure 2B), the decrease in the short interval was only observed after the R(+)-Baclofen treatment (*p* < 0.01). No differences were observed for the last IRI (20, ∞) between the 4 treatment groups.

In a previous study (Echeverry-Alzate et al., 2020), we showed that, within a same group, behavioral responses can be very different from one individual to another. We thus analyzed the variation of alcohol consumed and categorized them into 3 groups: 1. no change meaning that the variation in alcohol self-administration was lower than 10% from the Saline; 2. decrease for the rats who showed a decrease of at least 10% from baseline; and finally, 3. increase, for the rats showing an increase of more than 10% of their baseline self-administration (Figure 2D). We found that for the RS(±)-Baclofen treatment, 14 of 15 male rats showed a decrease in alcohol self-administration with an amplitude of −87.2% while in the females rats only 10 showed a decrease for an average amplitude of −73.7%. In both groups we found few rats showing an increase (1 for the males: +73%; 3 for the females: +96.8%). The R(+)-Baclofen seemed more effective since the 15 males and 12 females showed a decrease (−96.5% and −90.1% respectively). Regarding S(−)-Baclofen, the results were close between the males and the females rats with 7 males and 8 females showing an increase in alcohol self-administration (+70.5 and +113.8% respectively), 5 males and 6 Females exhibiting a decrease (−44.7% and −54.6% respectively).

### 3.2 RS(±)-Baclofen’s blood concentration and efficacy

**FIGURE 3:**
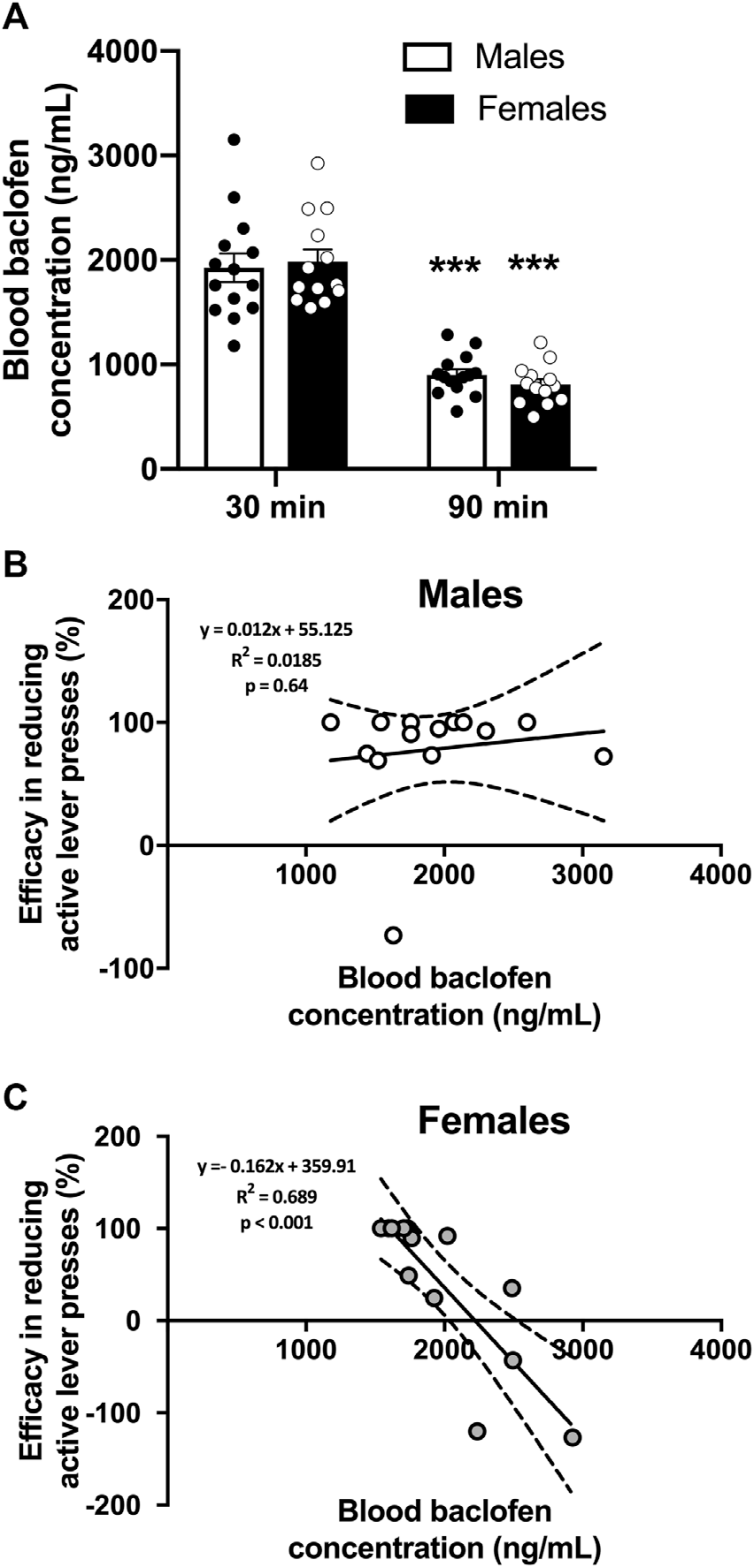
Blood RS(±)-Baclofen concentrations and correlation to its efficacy. **(A)** Blood samples were collected 30 and 90 min after the i.p. administration of a dose of 1.5 mg/kg of RS(±)-Baclofen. Results are expressed as Mean ± SEM blood Baclofen concentration in ng/mL. ***p < 0.001 vs. 30-min data within each sex. **(B,C)** Correlations between blood Baclofen concentration and efficacy in reducing alcohol operant self-administration in males **(B)** and females **(C)**. Blood Baclofen concentrations are expressed in ng/mL. Positive variations indicate an efficacy in reducing self-administration, negative numbers indicate opposite effect, meaning an increase in alcohol self-administration. For **(A**–**C)** males *n* = 14; females *n* = 13.

Blood samples were collected 30 and 90 min after RS(±)-Baclofen (1.5 mg/kg) was i.p. injected in the same animals of the previous experiment. We found that RS(±)-Baclofen was similarly metabolized in males and females (Figure 3A). However, the efficacy of RS(±)-Baclofen was independent of its blood concentration in males while it was inversely correlated in females with an increase in alcohol self-administration at the highest RS(±)-Baclofen’s blood concentrations. The concentration of Baclofen in the blood was measured by HPLC and the data analyzed using a 2-way RM ANOVA. Three samples were excluded because the detected levels were below the detection threshold. This analysis revealed an effect of the factor timepoint (F_(1,25)_ = 290.75, *p* < 0.001) but not of the factor sex (F_(1,25)_ = 0.02, ns) and no interaction between these factors (F_(1,25)_ = 1.37, ns). The Tukey test indicated a significant difference between the timepoints 30 and 90 min for both sexes (*p* < 0.001).

Then, we plotted the Baclofen concentration observed at the 30 min timepoint with the efficacy of the baclofen injection obtained from the previous experiment. The efficacy was calculated as the percentage of variation in the level of alcohol consumed between the RS(±)-Baclofen injection and the Saline injection. We found that there was no correlation (Pearson test, p = ns) between the blood Baclofen concentration in males (Figure 3B) whereas we observed a negative correlation (Pearson test, *p* < 0.001) in females (Figure 3C). In this latter group, we found that the highest Baclofen blood concentration resulted in an increase in alcohol self-administration while with lower baclofen concentrations the rats showed a decrease in alcohol self-administration.

### 3.3 Baclofen’s enantiomers metabolism

**FIGURE 4:**
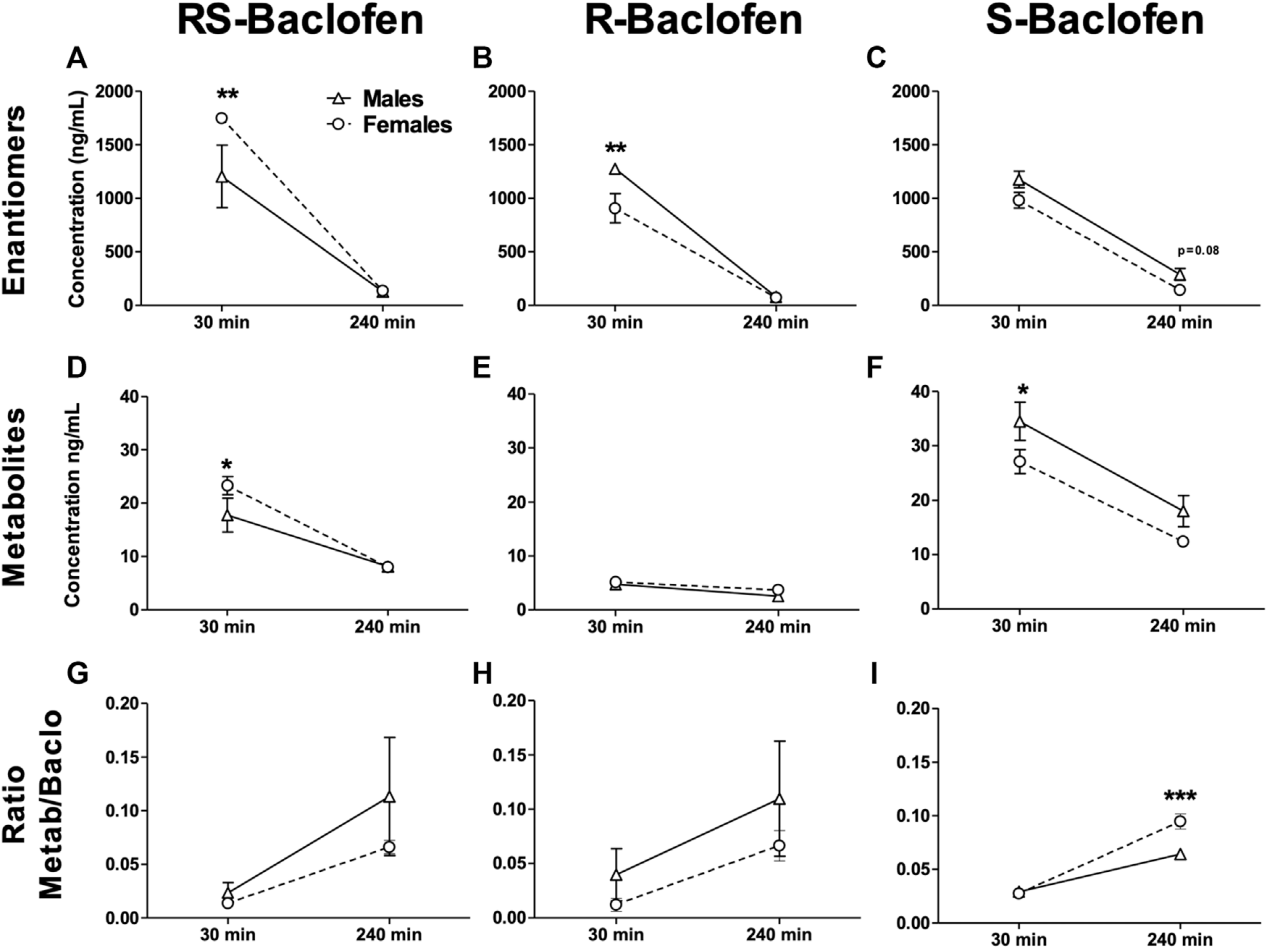
Metabolism of baclofen’s enantiomers in naïve males and female rats. The 3 Baclofen forms were i.p. injected randomly at the dose of 1.5 mg/kg and blood was collected 30 and 240 min after the administration. HPLC analysis was used to analyze the concentration of the enantiomers **(A**–**C)**, and the metabolite 3-(4-Chlorophenyl)-4-hydroxybutyric acid **(D**–**F)**. The ratio metabolite over enantiomers concentrations was then calculated **(G**–**I)**. Results are expressed as Mean ± SEM of blood concentrations in ng/mL. For **(A,D,G)** males *n* = 5; females *n* = 10. For **(B,E,H)** males *n* = 8; females *n* = 9. For **(C,F,I)** males *n* = 6; females *n* = 9. For clarity purpose we did not depict the significant differences we found for all the comparisons between the 2 timepoints within each sex group. *p < 0.05, **p < 0.01 and ***p < 0.001.

Naïve rats received each of the different enantiomers (1.5 mg/kg i.p.) at least 5 days apart. Thirty and 240 min after each injection, blood was collected, centrifuged and plasma analyzed to measure the concentrations of Baclofen and its metabolites. For all the forms of Baclofen administered, the profile was quite different at the timepoint 30 min with sometimes a higher concentration in the female group [RS(±)-Baclofen] and for the others, the opposite. At the second timepoint, 240 min post-injection, no clear sex difference was observed between the enantiomers. The detailed ANOVAs are compiled in Table 1. The multiple comparisons performed using the Tukey test are described below. For all the forms studied [different enantiomers and their metabolite: 3-(4-Chlorophenyl)-4-hydroxybutyric acid] and for the ratios, the Tukey test revealed a significant difference between both timepoints within each sex group (all p’s < 0.01). Some samples were excluded because the detected levels were below the detection threshold. In regards with sex groups analysis, the tests indicated a significant difference between males and females for the RS(±)-Baclofen (Figure 4A) and the R(+)-Baclofen (Figure 4B) for the timepoint 30 min (*p* < 0.01). Interestingly, for the RS(±)-Baclofen, the blood concentration was higher in the female group whereas for the R(+)-Baclofen it is the opposite. No differences were observed for the S(−)-Baclofen (Figure 4C). For all the 240 min timepoints, no differences were observed for any of the baclofen forms (Figures 4A–C). The level of the metabolites after the RS(±)-Baclofen injection was relatively low (Figure 4D) as compared to the Baclofen concentration suggesting a low level of metabolism and a high level of excretion. In regards with the R(+)-Baclofen, it is noteworthy that the levels of metabolites (Figure 4E) were, as expected (Sanchez-Ponce et al., 2012), even lower than previously observed with the racemic Baclofen (Figure 4D). At the 1st timepoint, sex differences were revealed by the *post hoc* for the racemic form of Baclofen (Figure 4D) and for the S(−)-Baclofen form (Figure 4F) with higher levels for the females groups with the racemic injection and the opposite with the S(−)-Baclofen (both p’s < 0.05). For the 3 forms of baclofen the levels of metabolites (Figures 4D–F) were also systematically significantly lower at the timepoint 240 min compared to the timepoint 30 min (all p’s < 0.01). Regarding the ratios (Figures 4G–I), no main effect was observed for the factor sex. An interaction between the factors sex and timepoint was revealed only for the S(−)-Baclofen (Table 1) with a significant difference between both sexes at the timepoint 240 min (Figure 4I) with the female group exhibiting a higher ratio metabolites/S(−)-Baclofen than the males.

**TABLE 1:** Statistical analysis of the blood concentration of the different baclofen enantiomers and their metabolites after an i.p. injection of a dose of 1.5 mg/kg at 30 and 240 min post-administration. Statistical analysis of the blood concentrations of the different forms of baclofen and their metabolites. The enantiomers and the racemic form were i.p. injected at a dose of 1.5 mg/kg in males and females, then blood was collected 30 and 240 min after the injection. Samples were analyzed by HPLC. Bold characters indicate a significant main effect and/or an interaction following a 2-way RM ANOVA.

Figure number	Factor name	F values	*p*-value
Figure 4A: Blood RS-Baclofen concentration in males and females	Sex	F_(1,13)_ = 3.82	*p* = 0.073
2-way RM ANOVA	**Timepoint**	F_(1,13)_ = 105.89	***p* < 0.001**
	Sex x Timepoint	F_(1,13)_ = 4.23	*p* = 0.060
Figure 4B: Blood Metabolite of RS-Baclofen concentration in males and females	Sex	F_(1,13)_ = 1.70	*p* = 0.215
2-way RM ANOVA	**Timepoint**	F_(1,13)_ = 67.54	***p* < 0.001**
	Sex x Timepoint	F_(1,13)_ = 3.38	*p* = 0.086
Figure 4C: Ratio Metabolites/RS-Baclofen blood concentration in males and females	Sex	F_(1,26)_ = 2.161	*p* = 0.154
2-way RM ANOVA	**Timepoint**	F_(1,26)_ = 13.304	***p* < 0.001**
	Sex x Timepoint	F_(1,26)_ = 0.907	*p* = 0.35
Figure 4D: Blood R-Baclofen concentration in males and females	**Sex**	F_(1,12)_ = 6.37	***p* < 0.05**
2-way RM ANOVA	**Timepoint**	F_(1,12)_ = 208.35	***p* < 0.001**
	**Sex x Timepoint**	F_(1,12)_ = 6.62	***p* < 0.05**
Figure 4E: Blood Metabolite of R-Baclofen concentration in males and females	Sex	F_(1,15)_ = 1.26	*p* = 0.28
2-way RM ANOVA	Timepoint	F_(1,15)_ = 16.99	***p* < 0.001**
	Sex x Timepoint	F_(1,15)_ = 0.67	*p* = 0.427
Figure 4F: Ratio Metabolites/R-Baclofen blood concentration in males and females	Sex	F_(1,30)_ = 1.579	*p* = 0.219
2-way RM ANOVA	**Timepoint**	F_(1,30)_ = 4.834	***p* < 0.05**
	Sex x Timepoint	F_(1,30)_ = 0.076	*p* = 0.785
Figure 4G: Blood S-Baclofen concentration in males and females	**Sex**	F_(1,13)_ = 7.706	***p* < 0.05**
2-way RM ANOVA	**Timepoint**	F_(1,13)_ = 233.36	***p* < 0.001**
	Sex x Timepoint	F_(1,13)_ = 0.149	*p* = 0.706
Figure 4H: Blood Metabolite of S-Baclofen concentration in males and females	**Sex**	F_(1,13)_ = 6.37	***p* < 0.05**
2-way RM ANOVA	**Timepoint**	F_(1,13)_ = 59.47	***p* < 0.001**
	Sex x Timepoint	F_(1,13)_ = 0.213	*p* = 0.652
Figure 4I: Ratio Metabolites/S-Baclofen blood concentration in males and females	**Sex**	F_(1,26)_ = 10.104	***p* < 0.01**
2-way RM ANOVA	**Timepoint**	F_(1,26)_ = 128.23	***p* < 0.001**
	**Sex x Timepoint**	F_(1,26)_ = 12.784	***p* < 0.001**
Blood concentrations of RS-Baclofen after a dose of 3 mg/kg in males and females	Sex	F_(1,12)_ = 0.090	*p* = 0.77
2-way RM ANOVA	**Timepoint**	F_(1,12)_ = 1041.80	***p* < 0.001**
	Sex x Timepoint	F_(1,12)_ = 0.038	*p* = 0.849

The RS(±)-Baclofen was also injected at the dose of 3 mg/kg and we found about twice the concentration than for the 1.5 mg/kg dose (2,978.57 ± 138.61 and 2,967.14 ± 112.52 ng/mL at the 30 min timepoint, 235.14 ± 15.76 and 190.43 ± 7.48 ng/mL for the 240 timepoint for males and females respectively). No difference between sex groups were observed.

### 3.4 Baclofen’s regulation of evoked dopamine release within the NAcc

**FIGURE 5:**
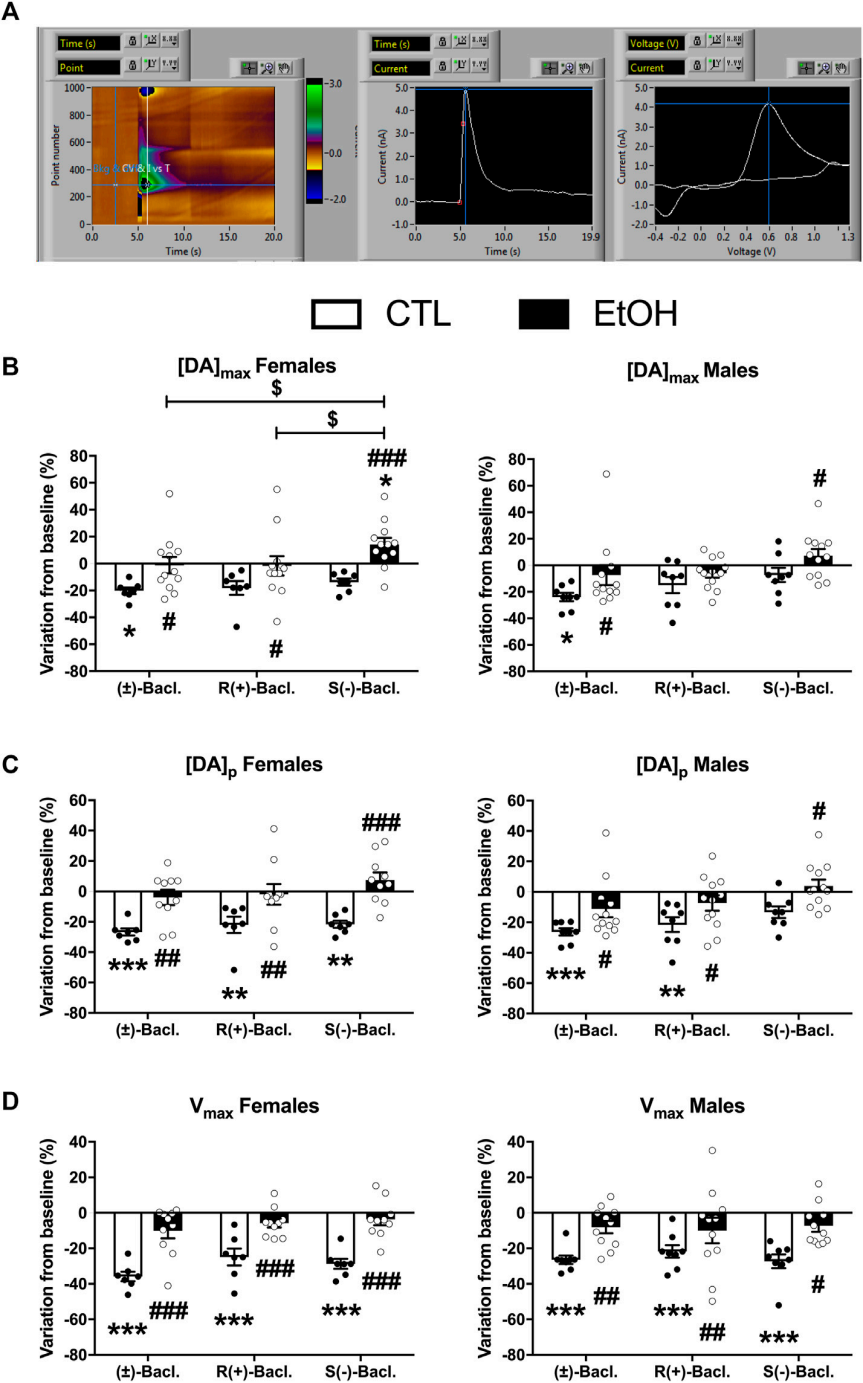
Effect of the different forms of Baclofen (100 µM) on phasic dopaminergic transmission in the nucleus accumbens core in male and female rats and in both control and alcohol groups. **(A)** Characteristic voltamogramm of dopamine, recorded currents. **(B–D)** Effect of different forms of Baclofen on [DA]_max_, [DA]_p_ and V_max_ in both male and female rats, respectively. Results are expressed as mean ± SEM of variation from baseline (%).

Baseline levels of the 3 parameters were not changed by chronic alcohol self-administration except the [DA]p that was significantly decreased in females (Supplementary Figure S3). The 3 forms of Baclofen were tested vs. baseline at the dose of 100 µM in FSCV, and the results are depicted in Figure 5. Within the group of alcohol-naïve rats, we found a decrease of the DA concentrations induced by the RS(±)-, R(+)- and S(−)-Baclofen in both males and females. In alcohol-exposed group, we observed a small decrease (not significant) of the DA levels in males induced by the R(+)- and RS(±)-Baclofen whereas an increase in DA release was observed with the S(−)-Baclofen. In females, the R(+) and the RS(±) forms had no effect on the evoked-DA levels but the S(−)-Baclofen also increased the DA release. See details of the statistical analysis for [DA]max, [DA]p and Vmax in Figure 3 and Table 2.

**TABLE 2:** Statistical analysis of the Baclofen’s regulation of evoked dopamine release within the NAcc, presented on Figure 4. Statistical analysis of the Baclofen’s regulation of evoked dopamine release within the NAcc. Bold characters indicate a significant main effect and/or an interaction following a 2-way RM ANOVA.

Figure panel	Factor name	F values	*p*-value
[DA]_max_: female rats (Figure 4B, left panel)	**Alcohol exposure slice treatment**	**F(1,51) = 9.42**	***p* < 0.01**
2-way RM ANOVA	**interaction**	**F(3,51) = 3.79**	***p* < 0.05**
		**F(3,51) = 3.54**	***p* < 0.05**
[DA]_max_: male rats (Figure 4B, right panel)	**Alcohol exposure slice treatment**	**F(1,54) = 6.86**	***p* < 0.05**
2-way RM ANOVA	interaction	**F(3,54) = 5.5**	***p* < 0.01**
		F(3,54) = 1.24	ns
[DA]_p_: female rats (Figure 4C, left panel)	**Alcohol exposure slice treatment**	**F(1,45) = 19.28**	***p* < 0.001**
2-way RM ANOVA	**interaction**	**F(3,45) = 5.24**	***p* < 0.01**
		**F(3,45) = 4.67**	***p* < 0.01**
[DA]_p_: male rats (Figure 4C, right panel)	**Alcohol exposure slice treatment**	**F(1,51) = 9.81**	***p* < 0.01**
2-way RM ANOVA	interaction	**F(3,51) = 8.69**	***p* < 0.001**
		F(3,51) = 1.84	ns
V_max_: female rats (Figure 4D, left panel)	**Alcohol exposure slice treatment**	**F(1,45) = 62.55**	***p* < 0.001**
2-way RM ANOVA	**interaction**	**F(3,45) = 19.02**	***p* < 0.001**
		**F(3,45) = 7.33**	***p* < 0.001**
V_max_: male rats (Figure 4D, right panel)	**Alcohol exposure slice treatment**	**F(1,51) = 9.96**	***p* < 0.01**
2-way RM ANOVA	**interaction**	**F(3,51) = 13.25**	***p* < 0.001**
		**F(3,51) = 3.27**	***p* < 0.05**

Because a correlation is expected between the peak of dopamine concentration and the V_max_ values, we checked the correlation at baseline and also after treatment with the different forms of Baclofen. We observed the expected correlations at baseline (data not shown) and the different forms of Baclofen did not change the correlations (Supplementary Figure S4).

## 4 Discussion

### 4.1 Effects on alcohol self-administration

A first cohort of animals self-administering alcohol in a binge drinking pattern was tested with a full dose response analysis in which all rats received all doses of the different forms of Baclofen in a sequential order [R(+) > S(−) > RS(±)]. Our results showed dose-responses for RS(±)-Baclofen and R(+)-Baclofen and revealed that females seemed less sensitive than males for R(+)-Baclofen effects. Results on males and R(+)-Baclofen show that the dose-response was consistent with our previous study (González-Marín et al., 2018). We also previously showed that R(+)-Baclofen (both 1 and 2 mg/kg doses) was more effective than S(−)-Baclofen in alcohol dependent rats (Echeverry-Alzate et al., 2020). Since a within-subjects design has been used in the present study, a possible interactive effect between treatments cannot be ruled out and thus it may explain why the sensitivity to the RS(±)-Baclofen seemed reduced in the first cohort of animals compared with the sensitivity to single 1.5 mg/kg dose tested in the second cohort. It should be pointed out that between-subjects design is more conservative while within-subjects design has more power.

To deeply analyze individual responses, we tested the different Baclofen forms in another cohort of animals using the 1.5 mg/kg dose and we showed that both the racemic form and the R(+)-Baclofen were always more effective in males than in females. Moreover, we demonstrated here that the racemic form, at this dose, was not effective in reducing alcohol self-administration in female rats. Regarding S(−)-Baclofen, the average alcohol self-administration was not altered by its administration in either males or in females. Overall, regarding the number of rats and the amplitude of increase of alcohol self-administration, whatever the enantiomer, females displayed the highest amplitude of change. The bimodal effect of both RS(±)- or R(+)-Baclofen enantiomers in females, with many rats reducing their intake but also several ones remaining engaged in alcohol intake may reveal lower response to the effect of Baclofen to reduce alcohol intake. It is quite unlikely that this difference may be due to motor side effects because we previously showed a significant reduction in locomotion starting at 2 mg/kg R(+)-Baclofen in male rats and no locomotor effects have been reported in females up to the 3 m/kg dose of RS(±)-Baclofen (González-Marín et al., 2018; Haile et al., 2021). The effect of the S(−)-Baclofen was even more complicated to summarize since about half of the group, both in males and females, exhibited a decrease in alcohol self-administration and the other half showed no change or an increase in alcohol self-administration. A very recent study in Sprague-Dawley rats with 10% alcohol operant self-administration sessions for 60 min, suggested that Baclofen (0.3–3 mg/kg, i.p.) was more effective in female rats (females: 42% and 58% decrease, males: 0% and 54% decrease in the number of lever presses after 0.3 and 1 mg/kg Baclofen, respectively) (Haile et al., 2021). The latter study also indicated that the 3 mg/kg dose was sedative in males and that hyperlocomotion was observed in females at the 1 mg/kg dose after repeated treatment (4 consecutive days). It should be noted, however, that this study did not clearly indicate that it used RS(±)-Baclofen and did not directly compare males and females in its statistical analyses.

### 4.2 Cumulative responses and motivation

The analysis of the cumulative lever presses responding revealed more strikingly the differences between the male and the female groups. The pattern of self-administration was strongly altered in males with the RS(±)- and the R(+)-Baclofen while it was more moderately affected in female self-administration and keeping the same shape of the response curve with only a lower starting point. Moreover, our results showed that RS(±)- and R(+)-Baclofen reduced alcohol self-administration at the very beginning of the sessions and this may indicate a mechanism in which Baclofen was effective even before the beginning of the session, thus could affect the motivation to consume alcohol. In addition, as mentioned in previous study (Colombo and Gessa, 2018), evaluating the pattern of self-administration with the cumulative data also indicated a reduction in lever pressing at the beginning of the session after intra-VTA micro-infusion of Baclofen. In addition, we found that there was a rightward shift of the inter-response intervals curves for the RS(±) and the R(+) forms of Baclofen only in the males whereas this rightward shift was only observed with the R(+)-Baclofen in females. Thus, our data are in line with those of the few studies evaluating the effect of Baclofen on motivation using a previous study progressive ratio paradigm and that showed a reduction in breaking point (an index of motivation) induced by Baclofen (Walker and Koob, 2007; Maccioni et al., 2008; Maccioni et al., 2012) [for review see (Colombo and Gessa, 2018)]. In this regard, different mechanisms have been suggested to explain the potential effectiveness of Baclofen in AUD. Baclofen may block the alcohol priming effect, may act as a partial substitution therapy or may modulate the responses to an initial drink (Chick and Nutt, 2012; Hauser et al., 2016).

### 4.3 Comparison with results from human studies

In humans, Baclofen has been found to be more effective in patients that consumed a larger number of drinks at baseline (Pierce et al., 2018). We previously showed in the post-dependent state model of AUD that the blood Baclofen levels were correlated to the efficacy to decrease alcohol self-administration in male rats, at the two tested doses of RS(±)-Baclofen (1 or 2 mg/kg) (Echeverry-Alzate et al., 2020). Here we did not find this correlation in male rats and this lack of correlation is very likely due to the high efficacy in males and a very low level of variability in the response to 1.5 mg/kg RS(±)-Baclofen. In females, the response variability was greater and individuals displayed an increase in self-administration. Thus, it seems that in general, preclinical studies were in favor of the dose response effect of Baclofen on alcohol self-administration, which is contrary to what is observed in clinical studies (Agabio et al., 2018; Garbutt et al., 2021). Here we clearly demonstrated a dose-response with R(+)-Baclofen. It is possible that in our previous study with post-dependent state animals, specific neuroadaptations in response to the alcohol vapors exposure would render some of the individuals less responsive to the Baclofen’s effect, thus, increasing the variability in response and the dose-response effect.

In contrast with the findings in males, we observed a negative correlation between the levels of blood RS(±)-Baclofen concentration and efficacy in reducing alcohol self-administration in the female group. Indeed, the higher the concentration was, the lesser efficacy was observed with a paradoxical increase of about 100% in the amount of alcohol consumed. To our knowledge, no previous preclinical data on the Baclofen’s efficacy were obtained in females and our results would need confirmation in human studies. This point could be critical in the clinical practice since we found that the RS(±)-Baclofen was less effective in females and may worsen misuse of alcohol with elevated concentrations of circulating Baclofen. Our results seem to contradict those of a previous preliminary open-label study on a very limited sample size (9 men and 3 women) suggesting that women were among the best responders regarding number of drinks per drinking day, number of heavy drinking days, and number of abstinent days (Flannery et al., 2004). However, these latter results must be tempered due to the very low sample size and the fact that more women were using antidepressant medication, making it difficult to determine whether the Baclofen or the Baclofen in combination with the antidepressant medication was helpful in drinking reduction (Flannery et al., 2004). Results from a very recent clinical study also suggested better responding in women. The study showed that sex was a moderator of response, with men benefiting from 90 mg of baclofen/day but not from 30 mg/day, whereas women showed benefit from baclofen 30 mg/day, marginal benefit from 90 mg/day, and worsened tolerability at 90 mg/day (Garbutt et al., 2021). Thus, women may be more responsive to low doses of Baclofen but may also be more susceptible to adverse drug reactions. A recent study also suggested sex as a potential moderator for Baclofen response in AUD since Baclofen significantly delayed the time to lapse for women but not male participants (Morley et al., 2022).

### 4.4 Blood Baclofen and metabolites levels

No sex difference was observed in blood Baclofen levels at both 30 and 90 min after injection of RS(±)-Baclofen (1.5 mg/kg) in rats chronically self-administering alcohol. Interestingly, in a separate cohort of rats naive for alcohol, the blood Baclofen concentrations 30 min after an identical injection of 1.5 mg/kg of RS(±)-Baclofen were higher in female than in male rats suggesting a modification of the distribution/metabolism properties induced by prolonged alcohol self-administration. In contrast, the R(+)-Baclofen concentration was higher in males than in females at the 1st timepoint post-injection. Human studies have demonstrated a higher sensitivity to side effects induced by RS(±)-Baclofen in women (Rigal et al., 2015). This suggest a differential mechanism of distribution of the different enantiomers and when both enantiomers are present together. This point emphasizes the importance of focusing the future research on the R(+)-Baclofen enantiomer and less on the racemic form. Concerning the Baclofen metabolites, our findings reveal that the blood levels of the S-Baclofen metabolites were 6 times higher than that of the R(+)-Baclofen after injection of each enantiomer separately and this result is in accordance with previous data in humans (Sanchez-Ponce et al., 2012). The level of the S(−)-Baclofen metabolite was even higher than the level of the RS(±)-Baclofen metabolite (at the same dose of 1.5 mg/kg), thus indicating a metabolic difference between enantiomers. Pharmacokinetic parameters of the R(+)- and S(−)-Baclofen enantiomers after a 20 mg oral dose of baclofen to normal human volunteers indicated similar plasma elimination half-lives (5.3 and 5.1 h respectively) but a slightly higher urinary excretion of R(+)-Baclofen relative to S(−)-Baclofen (Mutschler et al., 1990).

### 4.5 Mechanism of action of Baclofen of the different enantiomers

R(+)-Baclofen is more potent than S(−)-Baclofen (Witczuk et al., 1980; Falch et al., 2006). At the pharmacological level, R(+)-Baclofen and RS(±)-Baclofen may result in different behavioral outcomes because S(−)-Baclofen without being itself active, especially at a low dose, may increase or decrease the response to R(+)-Baclofen (Olpe et al., 1978; Fromm et al., 1990). A stereo-selective metabolic difference between R(+)- and S(−)-Baclofen, with no metabolites for observed after oral administration of the R(+)-enantiomer, whereas an oxidative deamination metabolite was observed after the administration of the R(+)- and S(−)- mixture, in humans (Sanchez-Ponce et al., 2012). Pharmacokinetic cannot explain the differences between enantiomers since the R(+)-Baclofen dose-dependently decreased ethanol self-administration, whereas a high S(−)-Baclofen dose increased ethanol self-administration when injected directly into the nucleus accumbens shell of C57Bl/6J mice (Kasten and Boehm, 2014). These latter results seem in line with our results since we also observed a decrease in alcohol self-administration after R(+)-enantiomer treatment and an increase in a significant proportion of animals after S(−)-Baclofen treatment, especially in females. A difference in the binding affinity of the two enantiomers and the existence of low- and high-affinity GABA_B_ receptors with low-affinity sites more selective for S(−)-Baclofen may explain selective behavioural effects (Bowery et al., 1983; Kasten and Boehm, 2014). Different neurotransmitter targets could also be involved. R(+)-Baclofen may interact with non-gabaergic sites (Waddington and Cross, 1980), while S(−)-Baclofen may interact with norepinephrine as well (Karbon et al., 1984). Importantly, the different enantiomers may not reach or leave the central nervous system at the same rate/amount because of asymmetric transport rate by organic anion transporters at the blood brain barrier. In this regard, it is interesting to note that the transport of R(+)-Baclofen across the blood brain barrier has been shown to be 4 to 5 times higher than S(−)-Baclofen or the racemic form. Thus, difference in the stereoselective transport may be also of interest to explain the better efficacy of R(+)-Baclofen.

### 4.6 Dopamine release

Among the neurobiological mechanisms underlying the effect of Baclofen in AUD, inhibition of dopamine release within the mesolimbic reward pathway is thought to be crucial. A complete inhibition of DA neurons firing was reached at a concentration of 100 μM *in vitro,* the same dose that we used here in our FSCV study (Cruz et al., 2004). Preclinical drug studies have demonstrated that 100 μM R(+)-Baclofen induced a 25% inhibition of dopamine release in the core of the NAcc probably by inhibiting the release probability (Pitman et al., 2014). Here we showed that RS(±)-Baclofen decreased all parameters ([DA]_max_, [DA]_p_ and V_max_) in control animals and that this effect was lost in the alcohol group, regardless of sex. R(+)-Baclofen also decreased [DA]_p_ and V_max_ values in control animals but not in the alcohol group, regardless of sex. S(−)-Baclofen significantly decreased [DA]_p_ in control groups but only in females, while V_max_ was decreased in both sexes. Strikingly, [DA]_max_, was increased only in females of the alcohol group. The difference on [DA]_max_ without altering in the V_max_ parameter suggest a change in DA release and not in DA transporter activity.

Even if we did not find any significant correlation between the effect of the different enantiomers on DA transmission and the effect on alcohol self-administration (data not shown), it seems that in general, the different forms of Baclofen were less effective to inhibit dopaminergic release in brain slices from animals that have chronically self-administered alcohol. The latter results seem to fit with our previous data showing that Baclofen was less effective in reducing alcohol self-administration of alcohol-dependent animals (Echeverry-Alzate et al., 2020). More interestingly, our results suggested that the increase in alcohol self-administration by S(−)-Baclofen, more frequent in females, may be associated with the increased dopamine release observed specifically in the alcohol group.

It is important to note that our experiment on DA release was designed to only explore the potential differential effects of the different forms of Baclofen in the same rats that consumed chronically alcohol and have also been treated with Baclofen *in vivo*. The use of the *ex vivo* FSCV technique is biased because we only measured the regulatory mechanisms involving the DA terminals and not the ones involving the somatic bodies located in the VTA and which are also the target of Baclofen (use of slices with disrupted neurocircuitries). The comprehensive explanation of the mechanism would require similar experiments but *in vivo*. To the best of our knowledge very few studies investigated the effect of Baclofen on DA release depending on the Baclofen enantiomer. In 1978, Waldmeier and Maitre, showed that the increasing effect of Baclofen (20 mg/kg) on striatal dopamine and metabolites (DOPAC and HVA) in rats was due to its S(−) but not R(+) enantiomer (Waldmeier and Maitre, 1978).

## 5 Conclusion

In the present study, we provided new and original data demonstrating that R(+)-Baclofen was the most powerful enantiomer in reducing the amount of alcohol consumed in a chronic self-administration model with a binge pattern of access. More importantly, more non-responders to RS(±)-Baclofen and to R(+)-Baclofen were present in females suggesting a sex difference in the efficacy that needs to be taken into account before administrating Baclofen. Moreover, more females than males displayed an increase in the total amount of alcohol consumed after the administration of the racemic form of Baclofen. We also demonstrated that the different enantiomers and the racemic Baclofen can act directly on the dopaminergic terminals within the NAcc and we observed a tendency for a bidirectional effect of Baclofen depending on the enantiomer especially in females. More generally, as in some pathologies the R(+) enantiomer is the one that should be preferred to others [S(−)-Baclofen and RS(±)-Baclofen] in the AUD therapy. For example, the R(+) enantiomer but not S(−)-Baclofen has been shown to reverse some deficits in animal models of autism (Silverman et al., 2015).

## Data Availability

The raw data supporting the conclusion of this article will be made available by the authors, without undue reservation.
