# Supplementary figures and images for: Sex-related differences in the efficacy of Baclofen enantiomers on self-administered alcohol in a binge drinking pattern and dopamine release in the core of the nucleus accumbens
Source: Front Pharmacol. 2023 Mar 16;14:1146848. doi: 10.3389/fphar.2023.1146848 (PMC10060511; doi:10.3389/fphar.2023.1146848)

## Females

### (±)-baclofen

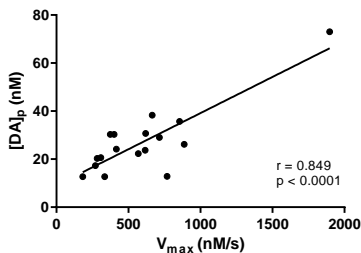

### R(+)-baclofen

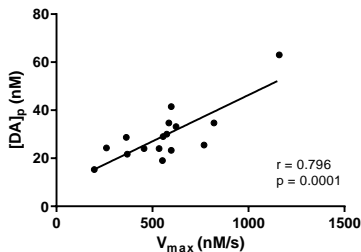

### S(-)-baclofen

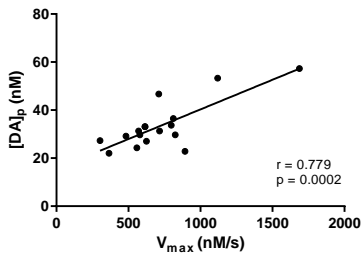

## Males

### (±)-baclofen

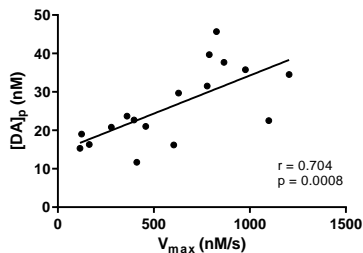

### R(+)-baclofen

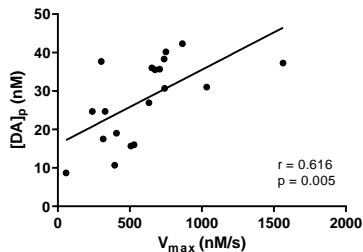

### S(-)-baclofen

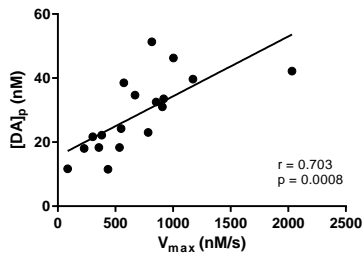

Supplement: Supplementary file 1 [file DataSheet2.PDF]

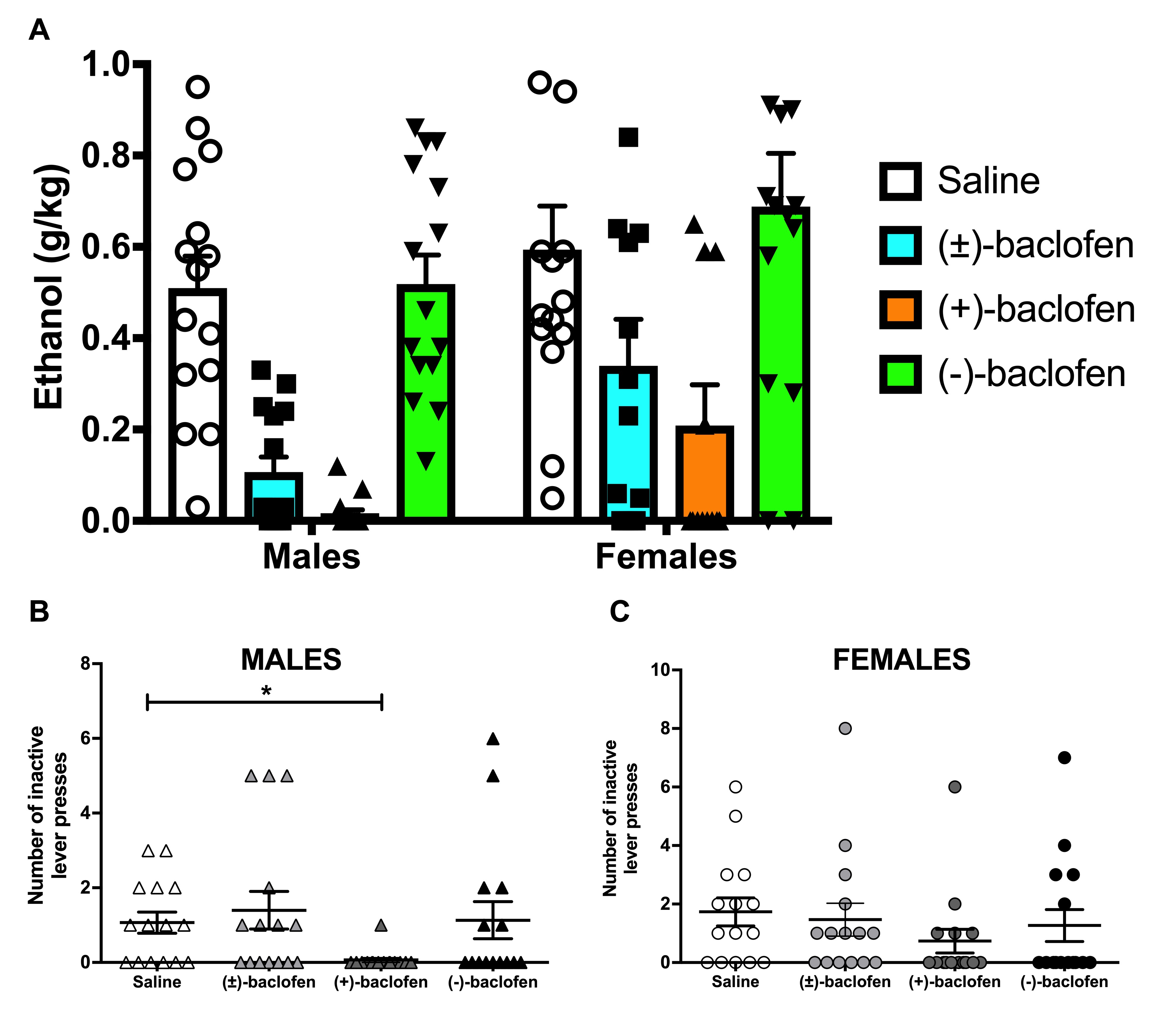

Supplement: Supplementary file 2 [file Image1.JPEG]

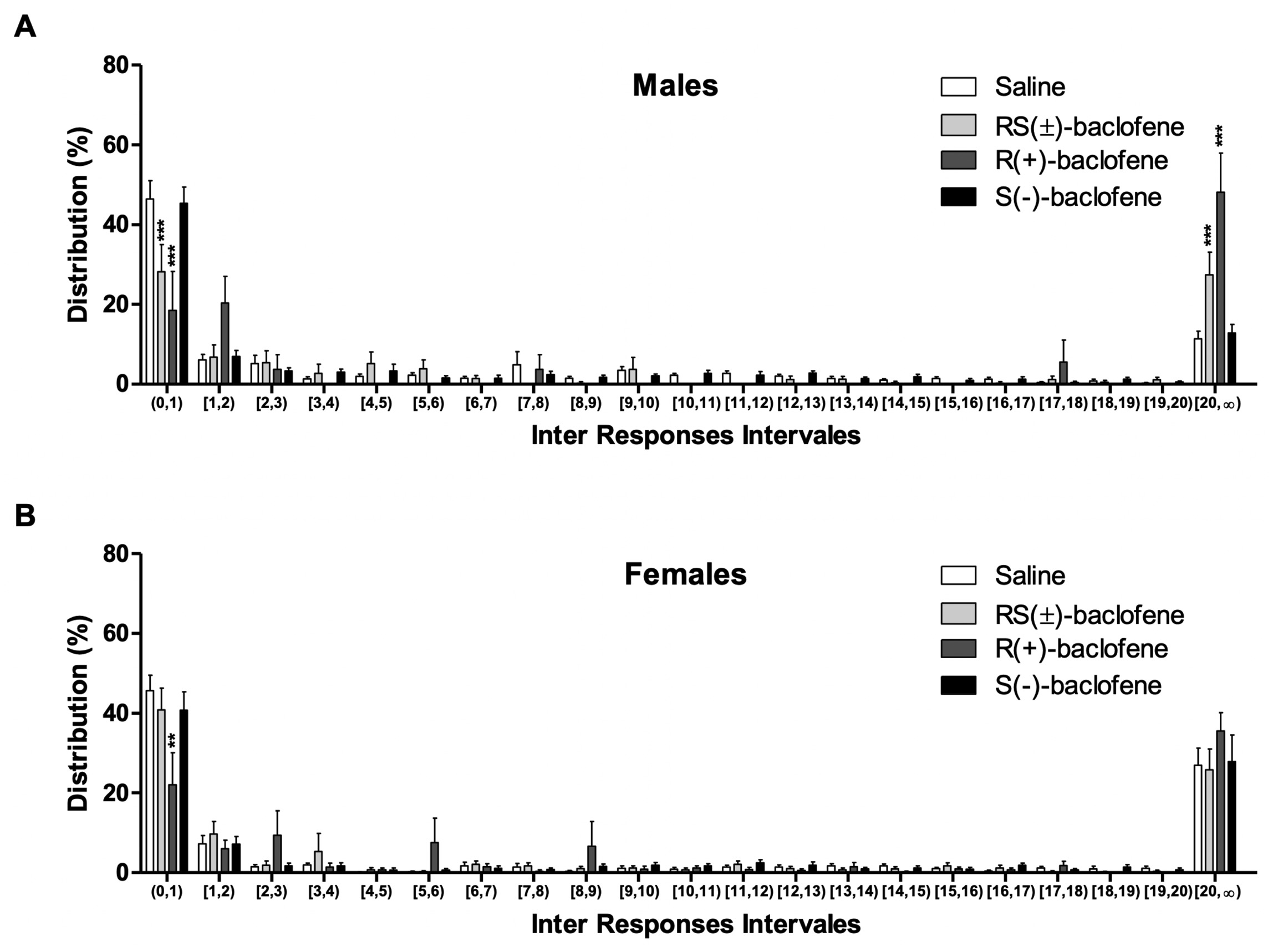

Supplement: Supplementary file 3 [file Image2.JPEG]

## Females

## Males

**A**

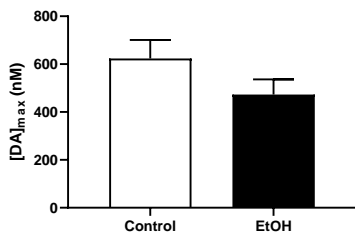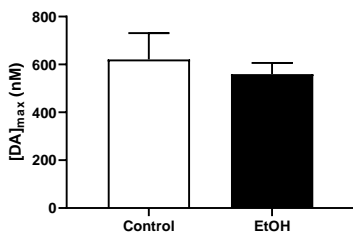

**B**

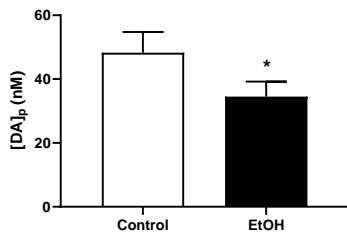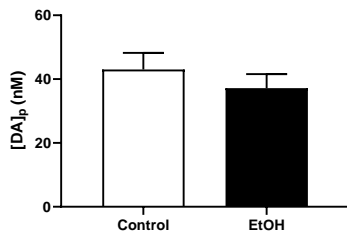

**C**

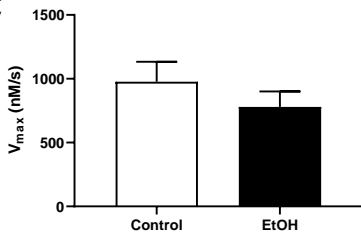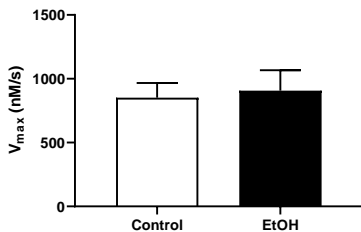

Supplement: Supplementary file 5 [file DataSheet1.PDF]
