# Supplementary material for: Sex-related differences in the efficacy of Baclofen enantiomers on self-administered alcohol in a binge drinking pattern and dopamine release in the core of the nucleus accumbens
Source: Front Pharmacol. 2023 Mar 16;14:1146848. doi: 10.3389/fphar.2023.1146848 (PMC10060511; doi:10.3389/fphar.2023.1146848)
Supplement: Supplementary file 4 [file DataSheet3.PDF]

Supplementary Informations

Results

Effects of the different baclofen enantiomers on ethanol self-administration

Compared to ethanol self-administration, results are similar when analyzed by the number of active lever presses in males (Supplemental Figure 1A) and in females (Supplemental Figure 1B). Regarding the inactive lever presses, only a moderate decrease is observed after the R(+)-Baclofen injection in the males (Supplemental Figure 1C). But the levels are already so low on average that this decrease is difficult to interpret. In the female group, no difference was observed for the numbers of inactive lever presses (Supplemental Figure 1D).

Legends of the supplemental Figures

Supplemental Figure 1: Effect of the different Baclofen enantiomers on ethanol operant self-administration in male and female rats: self-administered ethanol (g pure ethanol/kg body weight) and number of inactive lever presses. Self-administered ethanol depicted for the males and for the females (**A**) and expressed as Mean ± SEM. The analysis of the self-administered alcohol data (with a 2-way RM ANOVA revealed an effect of the factors sex ($F_{(1,28)} = 7.035$, $p < 0.05$) and treatment ($F_{(3,84)} = 20.27$, $p < 0.001$) and no interaction between these factors ($F_{(3,84)} = 0.34$, ns). Regarding the Number of inactive lever presses for the male group (**C**) the Kruskal-Wallis test revealed a main effect of the factor treatment ($K = 9.94$, $p < 0.01$) and the *post hoc* test indicated a significant difference between the vehicle and the R(+)-Baclofen (* $p < 0.05$). Concerning the females (**D**) the Kruskal-Wallis test did not reveal any main effect of the factor treatment ($K = 4.3$, ns). Results are expressed as Mean ± SEM. Males and females n = 15 each.

Supplemental Figure 2: Inter-response intervals in males (A) and females (B) after administration of the 3 forms of Baclofen. Data are obtained from the same test sessions performed for Figure 1 and 2. Rats were injected 30 prior to the test with a dose of 1.5 mg/kg of the RS(±)- ,the R(+)- and the S(-)-Baclofen. Inter-responses intervals are expressed as intervals of 1 sec except for the last one (20 sec to infinity). Results are expressed as Mean ± SEM percentage of total intervals. ** $p < 0.01$, *** $p < 0.001$.

Supplemental Figure 3: Baseline levels of the different parameters $[DA]_{max}$, $[DA]_p$ and V_{max} of the phasic dopaminergic transmission in the nucleus accumbens core in male and female rats and in both control and ethanol groups. A) $[DA]_{max}$, **B)** $[DA]_p$ and **C)** V_{max} in both male and female rats, respectively. Results are expressed as mean ± SEM. ** $p < 0.05$ (versus respective control group).

Supplemental Figure 4: Correlation between $[DA]_{max}$ and V_{max} in both males and females and in the whole population (control and ethanol groups). Significant correlation was observed in all groups and the different treatments did not change the correlation. Correlations were also observed during the baselines (before Baclofen treatments, data not shown).
